# Some Particulars in the Anatomy of the Whale

**Published:** 1800

**Authors:** John Abernethy


XX. *Some Particulars in the Anatomy of the Whale. By Mr.* John Abernethy, *F. R. S.* Vide *Philosophical Transactions of the Royal Society of London, for the Year* 1796. *Part I.* 4to. London, 1796.

THE parts which, in the whale, correfpond in fituation and office with the mefenteric glands of other animals, differ, it feems, confiderably from thofe glands in ftructure. Thefe peculiarities being not only curious in themfelves, but illuftrative of circumftances, hitherto deemed obfcure, in the anatomy and oeconomy of the lymphatic glands in general, Mr. Abernethy was induced to communicate an account of them to the Royal Society.

The animal, he tells us, from which the parts he has defcribed were taken, was a male of the genus named by Linnæus, *Balæna*.

Being defirous of making an anatomical preparation, to fhow the diftribution of the mefenteric veffels and lacteals of the whale, he procured for this purpofe a broad portion of the mesentery with the annexed inteftine;

 and and proceeded in the firſt place to inject the blood veſſels. The meſentery had been cut from the animal as cloſe to the ſpine as poſſible: had a leſs portion been taken away, he remarks, the parts he has deſcribed would have been left with the body, for they are ſituated upon the origin of the blood veſſels belonging to the inteſtines; and this, he thinks, may be the reaſon why they have not been obſerved before.

When he threw a red-coloured waxen injection into the meſenteric artery, he ſaw it meandering in the ramifications of that veſſel; but at the ſame time obſerved it collecting in ſeparate heaps, about the root of the meſentery, which ſoon increaſed to the ſize of eggs. At the time, he imagined that the veſſels had been ruptured, and that the injection in conſequence had become extravaſated.

He next threw ſome yellow injection into the vein, when ſimilar phænomena occurred; the branches of the vein were filled, but at the ſame time the maſſes of wax near the root of the meſentery were increaſed by a further effuſion of the injection. Theſe lumps, he obſerves, had now acquired a ſpherical form, and ſome of them were of the ſize of an orange.

After the injection had become cold, he cut into the mesentery, in order to remove these balls of wax; when he found that they were contained in bags, in which he also observed a slimy and bloody-coloured fluid. On the inner surface of these bags a great number of small arteries and veins were seen to terminate, from the mouths of which the injection had poured into their cavities. There were seven of these bags, it seems, in that piece of mesentery which he had to examine; but he is not able to determine what number belonged to the animal; as he does not know whether the portion of mesentery that he possessed was complete. Having removed the injection from these bags, he observed on the inside of them a soft whitish substance, apparently containing a plexus of lacteal vessels. This substance entered the bags at the part of them which was nearest to the intestines, and went out at the part next to the spine. He now poured some quicksilver into those lacteals which appeared to lead to this soft substance: the quicksilver in an instant entered the vessels which were contained in it, and thus its nature was ascertained. A number of lacteals having entered one of these bags, were observed ferved to communicate with each other, then again to feparate, and form other veffels, which went out of the bag. It was fome time before the quickfilver paffed through the plexus of veffels contained in the firft bag; but after having pervaded it, it paffed on to a fecond bag, in which was concealed a fimilar plexus of lacteals. The quickfilver permeated thefe laft veffels with much greater facility than it did the former, and quickly ran out of the large lacteals which were divided at the origin of the mefentery. Befides thofe abforbents which paffed through the bags in the manner defcribed, Mr. Abernethy obferved great numbers of others, which terminated by open orifices in every part of them. When quickfilver was poured into any of the lacteals, which were found near the fides of the bags, it immediately ran in a ftream into their cavities. He introduced about a dozen briftles through as many lacteals, into different parts of two of thefe bags. Thefe, he obferves, were few, in comparifon to the whole number which terminated in them, but as the mefentery was fat, and the veffels were fmall, more could not eafily be paffed.

He afterwards ftuffed two of the bags with horfe hair, dried them, and preferved them as an anatomical preparation. In this ftate, he tells us, great numbers of arteries and veins, but chiefly of the former veffels, are feen terminating on their infide in the fame indiftinct manner as the foramina Thebefii appear when the cavities of the heart are laid open: the briftles alfo, he obferves, render vifible the termination of a certain number of lacteals. He examined the fides of thefe bags, which were moderately thick and firm; but he did not fee any thing which, from its appearance, he could call a mufcular ftructure.

From the circumftances that have been related, it appears, obferves our author, that in the whale there are two ways by which the chyle can pafs from the inteftines into the thoracic duct; one of thefe is through thofe lacteals which pour the abforbed chyle into bags in which it receives an addition of animal fluids; the other is through thofe lacteals which form a plexus on the infide of the bags; through thefe veffels, he remarks, it paffes with fome difficulty, on account of their communications with each other; and it is conveyed veyed by them to the thoracic duct, in the same state that it was when first imbibed from the intestines. The lacteals, which pour the chyle into the bags, he finds similar to those which terminate in the cells of the mesenteric glands of other animals: there is also, he remarks, an analogy between the distribution of the lacteals on the inside of these bags, and that which is sometimes seen on the outside of the lymphatic glands in general. In either case, he observes, a certain number of the vasa inferentia, as they are termed, communicate with one another, and with other vessels, named vasa efferentia.

By this communication, Mr. Abernethy remarks, the progress of the fluids contained in these vessels is in some degree checked; and the impediment, he thinks, increases the effusion into the cavities of the gland made by the other lacteals: but should these cavities be obstructed from disease, or other causes, an increased determination of fluids into the communicating absorbents, he supposes, must happen, which would overcome the resistance produced by their mutual inosculations, and the contents of the vessels would be driven forwards towards the trunk of the system. In the whale, as in other animals, he finds that the impediment, occaſioned by this communication of lacteals, is greateſt in the firſt glands at which they arrive after having left the inteſtines.

From the ready termination of ſo many arteries in the meſenteric glands of the whale, Mr. Abernethy thinks it probable there is a copious ſecretion of fluids mixed with the abſorbed chyle; and this ſeems to account for the ſlimy bloody coloured fluid found in them. As the orifices of the veins were open, he is inclined to think that the contents of the bags might paſs in ſome degree into thoſe veſſels.

Albinus, Meckel, Hewſon and Wriſberg, he obſerves, have been of opinion, that the lymphatic glands are not cellular, but compoſed of convoluted abſorbing veſſels. This notion, however, he adds, appears to have been gradually declining.

Mr. Cruikſhank, it ſeems, has of late publicly maintained a contrary opinion; and has ſhewn, that the cells of theſe glands have tranſverſe communications with each other, which it is not likely they would have, if they were only the ſections of convoluted veſſels. Some additional obſervations have occurred to to our author, confirming this opinion, and which as they have not been publickly noticed by others, he has been induced to relate to the Society. He has injected the lymphatic glands of the groin and axilla of horfes, with wax, and afterwards deftroyed the animal fubftance, by immerfing them in muriatic acid. In fome of thefe glands the wax appeared in very fmall portions, and irregularly conjoined; which is a convincing proof, he thinks, that it had acquired this irregular form from having been impelled into numerous minute cells. But in feveral inftances, he found one folid lump of wax, after the deftruction of the animal fubftance: and it appears to him fufficiently clear, that the glands which were filled in this manner, were formed internally of one cavity, and were not, as is commonly the cafe, compofed of many minute cells. He has alfo, he obferves, filled glands of this ftructure in the mefentery of an horfe, with quickfilver: he has then dried them, cut open the bags, and introduced a briftle into them through the vas inferens. And in the human mefentery, after having injected the artery, he has filled a bag refembling a gland, with quickfilver; which which being opened, a mixture of injection and quickſilver was found in its cavity.

That the lymphatic glands in moſt animals are cellular, may not, perhaps, our author thinks, be hereafter doubted: that they are ſometimes mere bags, analogy and actual obſervation induce him to believe. If it ſhould be ſaid, that in thoſe inſtances which he has related, the cells were burſt, or that the glands were diſeaſed; he can only, he obſerves, reply, that there was no appearance to lead him to ſuch a concluſion.

If, then, the lymphatic glands are either cellular, or receptacles reſembling bags for the abſorbed fluids, we are naturally led, he thinks, to inquire, what advantage ariſes from this temporary effuſion of the contents of the abſorbents. That there is a conſiderable quantity of fluids poured forth from the arteries of the whale, to mix with the abſorbed chyle, is, he remarks, very evident; nor can it be doubted, he thinks, that the ſame thing happens in other animals; the cells of the lymphatic glands being eaſily inflated, and injected from the arteries.

The ready communication of theſe bags with the veins of the whale, induced him, it ſeems, feems, to examine whether he could afcertain any thing fimilar in other animals. Air impelled into the lymphatic glands, however, he obferves, feldom gets into the veins; fometimes indeed, he has feen veins injected from thefe glands; but when this has occurred to him, he has obferved an abforbent arifing from the gland, and terminating in the adjacent vein.

